# Functional morphology and structural characteristics of the hind wings of the bamboo weevil *Cyrtotrachelus buqueti* (Coleoptera, Curculionidae)

**DOI:** 10.1080/19768354.2019.1592020

**Published:** 2019-03-24

**Authors:** Xin Li, Ce Guo, Longhai Li

**Affiliations:** a College of Mechanical and Electrical Engineering, Nanjing University of Aeronautics and Astronautics, Nanjing, People’s Republic of China; b Institute of Bio-inspired Structure and Surface Engineering, Nanjing University of Aeronautics and Astronautics, Nanjing, People’s Republic of China

**Keywords:** Hind wings, scanning electron microscopy, aerodynamics, resilin, mechanical properties

## Abstract

Research data of the microstructure and surface morphology of insect wings have been used to help design micro air vehicles (MAV) and coating materials. The present study aimed to examine the microstructure and morphology of the hind wings of *Cyrtotrachelus buqueti* using inverted fluorescence microscopy (IFM), scanning electron microscopy (SEM), and a mechanical testing system. IFM was used to investigate the distribution of resilin in the hind wing, and SEM was performed to assess the functional characteristics and cross-sectional microstructure of the wings. Moreover, mechanical properties regarding the intersecting location of folding lines and the bending zone (BZ) were examined. Resilin, a rubber-like protein, was found in several mobile joints and in veins walls that are connected to the wing membranes. Taken together, structural data, unfolding motions, and results of tensile testing suggest two conclusions on resilin in the hind wing of *C. buqueti*: firstly, the resilin distribution is likely associated with specific folding mechanisms of the hind wings, and secondly, resilin occurs at positions where additional elasticity is needed, such as in the bending zone, in order to prevent structural damage during repeated folding and unfolding of the hind wings. The functional significance of resilin joints may shed light on the evolutionary relationship between morphological and structural hind wing properties.

## 1. Introduction

Wings of flying insects have a high degree of accuracy and flexibility, which is far superior to the MAV with a low Reynolds number designed by humans to imitate their flight capabilities. With the changing nature of the environment, the flying skills of insects are also evolving. Coleopteran insects’ fore wings are often both thickened and stiff, which can prevent their hind wings from being destroyed during ground locomotion and can reduce their abdomen's motility during flight. Additionally, the flexible wing of insects has a kind of complex venation structure that can control the torsion under the aerodynamic forces, which is also the main reason that the size of the hind wings is larger than that of elytra. Previous studies have shown that insects in flight adjust their flight attitudes through the thorax muscles to distort the wing base, and no muscle exists in the wing membranes and veins, instead of resilin is located in some specific areas (Hass et al. 2000a, 2000b; Nguyen et al. 2010; Ma et al. 2017).

How do beetles fold and unfold their hind wings? Since the question was raised (Forbes 1924), many researchers have conducted a series of in-depth investigation on the folding and deployment mechanisms of the hind wings of insects such as earwig (Spongiphoridae and Forficulidae), the beetle *Pachnoda marginata* and the rove beetle *Cafius vestitus* (Hass et al. 2000a, 2000b; Saito et al. 2014; Deiters et al. 2016). The deployment movement of the earwig's hind wings is achieved through the thoracic musculature and the inherent strengthening mechanisms (Haas et al. 2012; Deiters et al. 2016), in which the legs and cerci are never involved (Haas et al. 2012). The hind wing folded and unfolded in a similar manner to the fans, folded twice in the transverse direction, which is a unique folding pattern in insects (Haas et al. 2000b), and the folding of hind wings is achieved by the elasticity of the storage. Some studies (Kukalová-Peck and Lawrence 1993; Brackenbury 1994; Haas & Beutel 2001) have observed that the unfolded pattern of the beetle's hind wings is a kind of typical deployable structure. During the folding process, the actuation of the muscles in the abdomen and the mesothorax triggers the radius anterior (RA) and the media posterior (MP) forms a scissors-like movement and allows them to move closer each other to drive the entire wing to be folded. During this whole period, the energy is stored in the elastic structure, which is formed by the edge of the wing veins, and the wing remains folded by the coupling apparatus of the wings. The RA and MP separated from each other because the contraction of a muscle in the thorax triggers a scissor-like movement and release of the elastic energy stored in resilin which results in the unfolding of the hind wings. There are also results showing that the folding of the ground-beetle's wings (Carabidae) is achieved by the fore wings, abdomen, and thorax muscles, and the unfolding is accomplished by the elastic mechanism of the wings (Hammond 1979). Until now, the exploration of the mechanism of folding and unfolding of the deployable flexible wing and the material properties of the insect's wing is still ongoing.

Reslin, a rubber-like protein, is an integral component of insect wings as it provides high elasticity and prevents structural damage after repeated folding and unfolding of the wings. Numerous studies have been conducted on the topic (reviewed in Donoughe et al. 2011), and it has been shown that resilin present in the vein-joints of dragonfly wings was crucial for the torque control during the beating of dragonfly wings and in response to aerodynamic effects (Gorb 1999). Another study revealed that odonate wings seem to adapt to reversible deformations so that the wing membranes and wing veins are not permanently damaged by external forces (Newman & Wootton 1986). Most elastic deformations of the flying wings of insects result from the passive mechanical properties of the beating wings and inertia forces and aerodynamics during flight (Wootton 1992; Daniel & Combes 2002). This flexibility helps to enhance the aerodynamics of wings, and the passive deformation mechanism is important for sustaining aerodynamic forces. For example, previous studies showed the compliance wings of the moth *Manduca sexta* (Mountcascle & Daniel 2009) and the desert locust *Schistocerca gregaria* (Young et al. 2009) are subjected to bending deformation but can generate a greater lifting force than rigid wings due to their elasticity.

Haas and his co-workers used a rectangular film to illustrate the working principle of the folding and unfolding of the *Diploptera punctata* cockroach's hind wing, which was defined as a four-plane mechanism (Hass & Wootton 1996). There are also some other insect wings whose folding principle follows the four-plane principle, such as the earwig *Forficula auricularia* (Haas et al. 2000b; Deiters et al. 2016), the *Allomyrina dichotoma* beetles (Muhammad et al. 2009), and *Priacma serrata* (Haas 2000). In order to be completely covered by the elytra when the insects landed, the hind wings of the *C. buqueti* are extensively folded two times transversely and longitudinally. The storage ratio **of** the hind wings of *C. buqueti* was calculated as 3 by using grid method, which is higher than the folding ratio of 2.3 in the *A. dichotoma* beetle's wings (Muhammad et al. 2009).

In the present study, the functional morphology and microstructures of hind wings of *C. buqueti* were examined using individuals collected in bamboo forests. The microstructure of the major veins, such as radius anterior (RA 3) and media posterior (MP 1+2) were examined in cross-sections using scanning electron microscopy, which showed that the veins were structured in layers. Furthermore, we assessed the mechanical properties in the locations of intersecting folding lines and the bending zone. This may indicate that the hind wings of *C. buqueti* have a special advantage that related to the design of bionic flapping-wing micro air vehicle. They include not only the small size and the ability to fold when not in use, but also inspiring engineers to design advanced composite materials.

## 2. Materials and methods

### 2.1. Sample preparation

**Figure 1.**
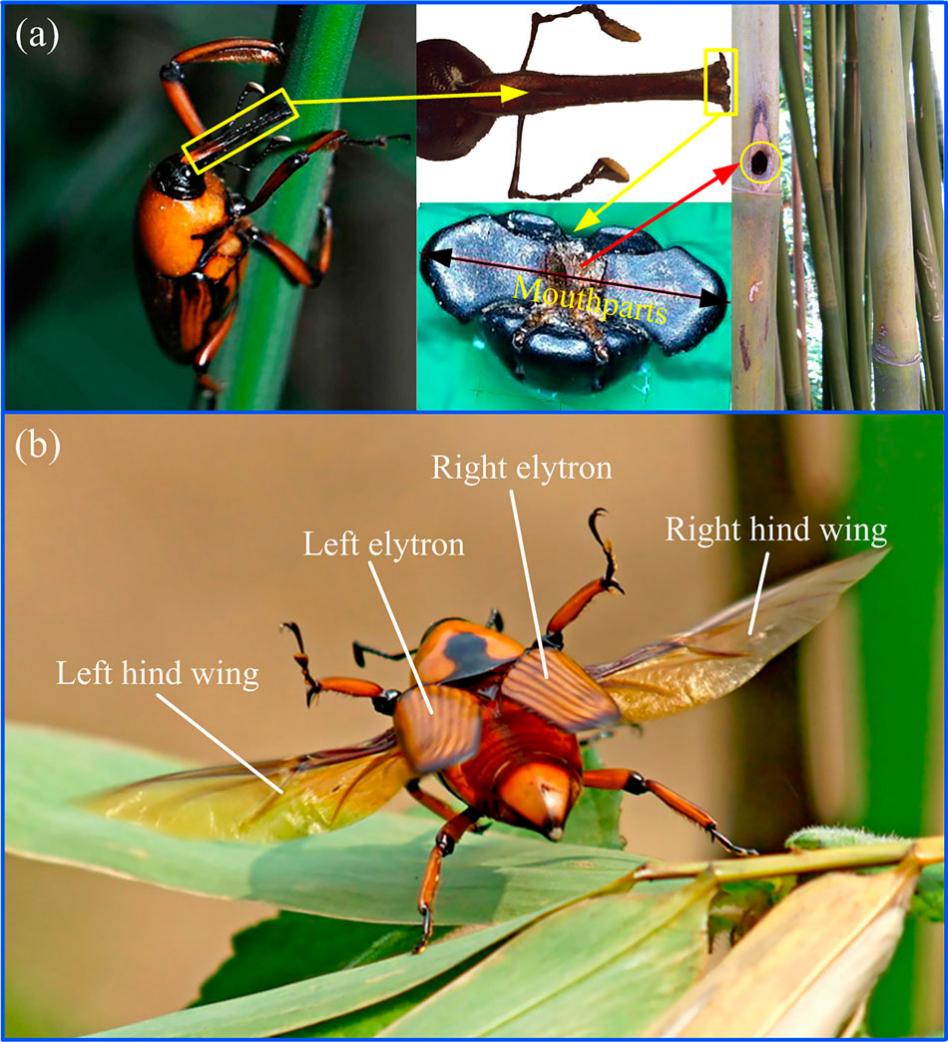
The collected specimens were from *Leshan* City, *Sichuan Province*. (a) A pair of elytra is closed, and the hind wings are folded under elytra. (b) The hind wings is fully unfolded.

Specimens of the bamboo weevil *Cyrtotrachelus buqueti* were used for morphological investigations of the hind wings (Figure 1). All specimens were obtained from *Leshan* City, *Sichuan province*, China, in the summer of 2016. Some of them were stored in the refrigerator prior to research and other specimens were fed with fresh bamboo shoots to prepare for high-speed photography. The weight of specimen was 3–4 g, and the length of a single hind wing was close to 43 mm. Before observation under a microscope, the hind wings of the specimens were fixed on the slide glass and kept at room temperature for 15 min so that moisture could be completely evaporated. Before inverted fluorescence microscope (IFM), the hind wings were washed using filtered phosphate-buffered saline (PBS) to remove dust and were stored at room temperature. For SEM was performed, the insects were dehydrated using 70% ethanol and were then placed in an incubator for at least 12 h. Drying may cause wrinkling of the wing surface, however, this process is important for obtaining high-quality images. After drying, the hind wings of each individual were removed for morphological examinations.

### 2.2. IFM observation

This method can reveal the presence of the intrinsic resilin, a peculiar protein, without the need for antibody labeling or other chemical treatments. In order to more directly observe the distributions of resilin in the hind wings of *C. buqueti*, the four dried hind wings were separately mounted between two coverslips with the anhydrous ethanol medium to flatten them. We observed the samples under an inverted fluorescence microscope (Eclipse Ti, Nikon, Japan) in one of three bands of wavelengths: the green (excitation wavelength of 510–560 nm, emission wavelength 590 nm), blue (excitation wavelength of 450–490 nm, emission wavelength 520 nm) and an ultraviolet ray (UV) band (excitation wavelength of 330–380 nm, emission wavelength 420 nm). We conducted a detailed investigation on the ventral and dorsal of each hind wing. The temperature and humidity in the laboratory kept at 25°C and 70%, respectively.

### 2.3. High-speed photography

Through long-term field observations and insect's behavior experiments, it was found that *C. buqueti* has strong flying ability. It not only can shuttle freely in the dense bamboo forest, but also can fly to the ground to drill and repair the nest. Therefore, in order to adapt to the change of the living environment quickly, the flying wings of *C. buqueti* play an irreplaceable role. In order to photograph the state changes of *C. buqueti* hind wings during deployment of the wings, a high-speed camera (Olympus, i-SPEED 3, Tokyo, Japan) was used to capture the video sequences of the hind wings unfolded at a speed of 2000 frames per second, which is the best tool for dynamic image acquisition and analysis.

### 2.4. Scanning electron microscopy

Due to the excellent flying capability of *C. buqueti*, high stability was expected. The wingspan is about 90–100 mm, which allows to provide sufficient lifting power and thrust during flight. The main veins (C, Sc, RA and MP) may play an important supporting role when the hind wings are subjected to bending deformation due to aerodynamic effects. According to previous studies (Herbert et al. 2000; Ren & Li 2013; Sun et al. 2016; Ma et al. 2017), vein cross-sections differ substantially between insect species, which considerably affects the mechanical properties. In the present study, the cross-sectional structures of the dried RA and MP were coated with gold-palladium and examined using SEM (Quanta200, FEI, USA) at an accelerating voltage of 20 kV. Before SEM, each major wing vein was cut into three parts using sharp blades, and were then fixed on a slide using conductive tape for practicality. Dust was removed using compressed air in order to prevent surface impurities from affecting the observations.

### 2.5. Mechanical testing of specimens

**Figure 2.**
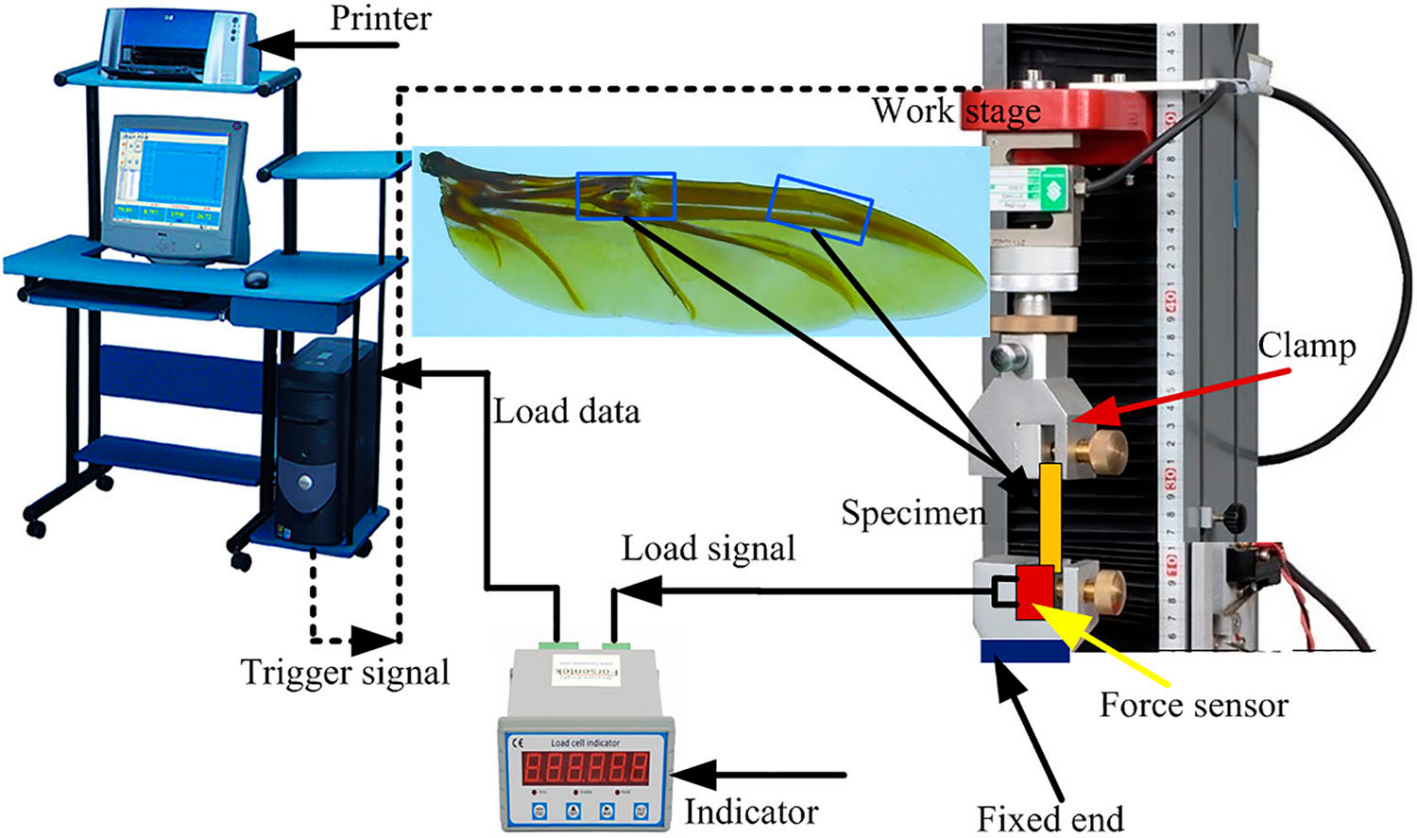
Overall tensile testing system used to measure the mechanical properties of the bending zone and the location of intersecting folding lines. The load signal from the force sensor is transferred to the control computer.

The mechanical properties of the intersections of the folding lines and the bending zone were obtained using a tensile testing system (CMT4503, China), as shown in Figure 2. These tests were conducted at the School of Materials Science and Engineering of Southeast University, China. According to previous studies (Song et al. 2004), the mechanical properties of wings change rapidly after removal from the insect; thus, care was taken to complete the testing of each sample within 15 min. For this reason, three samples were examined regarding the bending zone and the location of intersecting folding lines, respectively, and the total test time did not exceed 1 h. A load velocity of 1 mm/min was set to carry out the tests and the typical force versus displacement curves were obtained.

## 3. Results

### 3.1. Hind wing morphology

**Figure 3.**
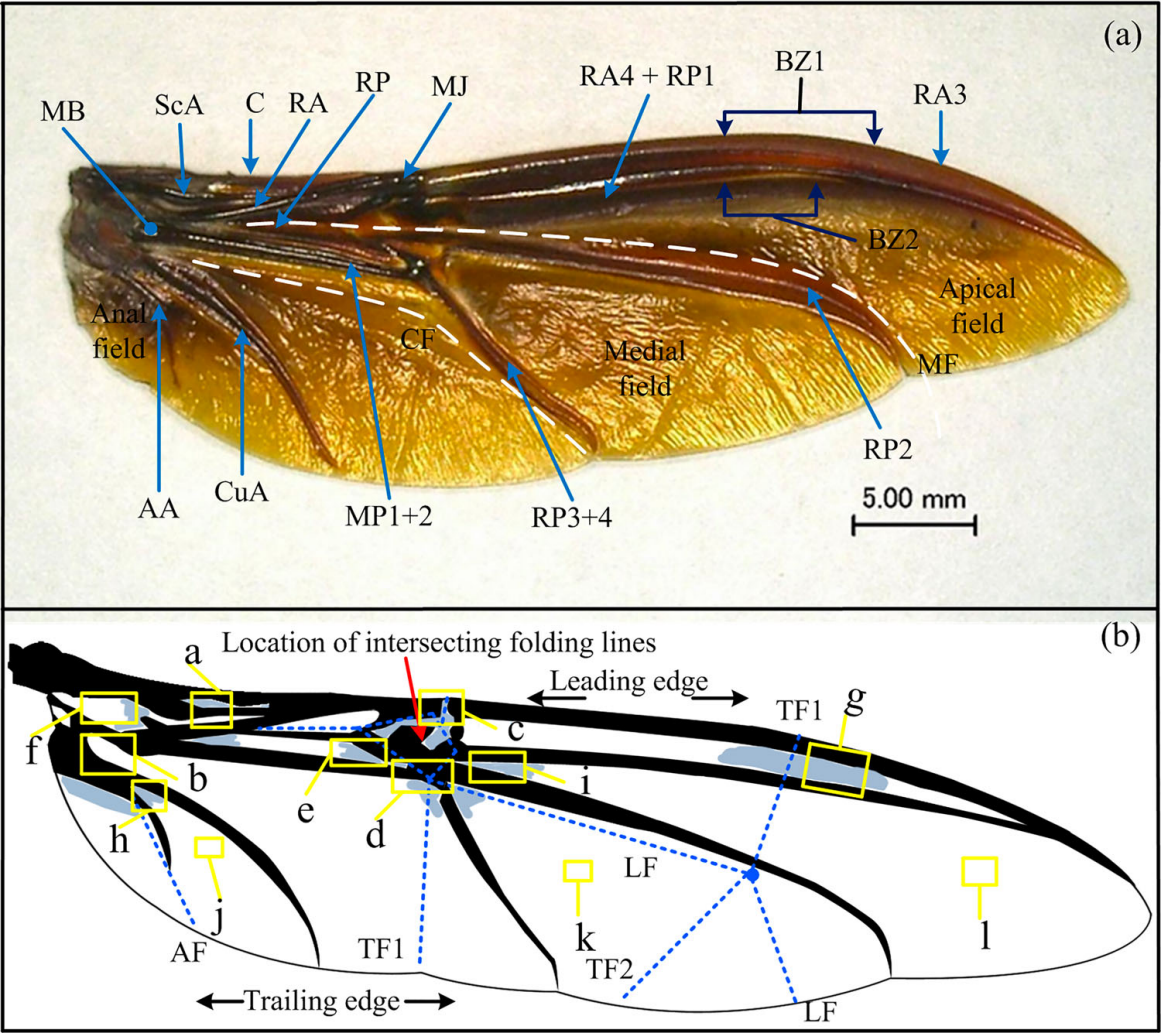
*C. buqueti*, a hind wing showing veins, resilin-containing areas, flexion and main folding lines. (a) The hind wing showing veins and flexion lines. (b) The hind wing showing areas with resilin and major folding lines. Twelve rectangles (a-l) indicate where the resilin probably present, as shown in Figure 4. C, costa; ScA, subcosta anterior; CuA, cubitus anterior; RA, radius anterior; RP, radius posterior; MP1+2, media posterior; AA, anal anterior; MB, medial bridge; MJ, marginal joint; BZ, bending zone; TF, transverse fold; LF, longitudinal fold; AF, anal fold; CF, claval flexion; MF, median flexion.

*Cyrtotrachelus buqueti* has two pairs of wings, and each pair includes an elytron and a hind wing (Figure 1). The whole shape of the unfolded wing is similar to the sail, which is supported by the longitudinal wing veins, and probably this feature is the reason why the insects endures a great bending deformations of the wing without destroying the wing during the flapping wing flight. Similar to the naming rules of other Coleoptera venation, the claval flexion line of *C. buqueti* is located near the MP and RP (Figure 3(b)). *C. buqueti* has simple horizontal and vertical folding techniques, which is simpler than dragonflies and earwigs (Donoughe et al. 2011; Deiters et al. 2016).

Slightly different from *A. dichotoma* beetles (Ha et al., 2013), the RA of *C. buqueti* was flexible and included two bending zones, BZ1 and BZ2 (Figure 3), and BZ1 and the marginal joint (MJ) were located at about one-third of the wing length. The vein MP1+2 terminated at the joint point, and the RP3+4 produced a convex formation with the vein MP1+2. The folding of the hind wings occurred based on two four-plane folds (Figure 8(b)), each of which shared two adjacent planes between them. The one most distant to the edge of the articulation, the first transverse fold (TF1), passed through the wing veins and ends at the tip of the RP3+4 and the bend zones. The proximal folds, the second transverse fold (TF2), crossed the wing and ended at the distal end of the vein MP1+2 and the marginal joint. The longitudinal folds (LF) originated at the middle bridge (MB) and had no branches at the intersection with the TF2. The median flexion line (MF) was located at the leading edge of the longitudinal fold and radiated along the vein RP2. The overall geometry of the folded wings of *C. buqueti* resembled a Z-shape, which was determined by the claval flexion (CF) line and the median flexion (MF) line. As confirmed by observations, the hind wings of *C. buqueti* are typically stowed under the elytra when folded, and rest on the thorax surface in a curved shape.

### 3.2. Resilin distribution in the hind wings of *C. buqueti*

**Figure 4.**
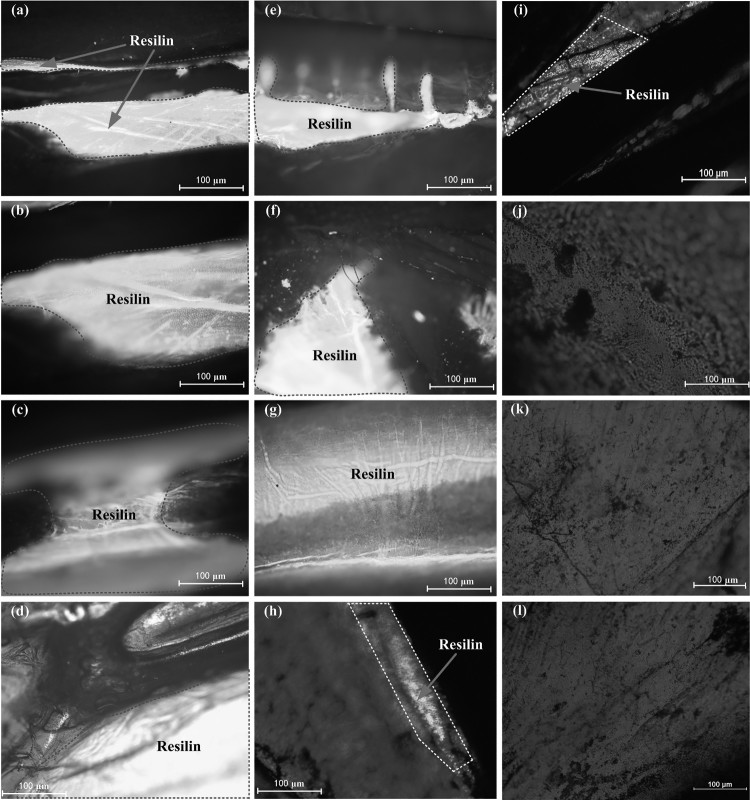
The distribution pattern of resilin in the hind wings of. *C. buqueti*. The bright dark-blue areas contain the rubber-like protein resilin. The regions of the selected, where the resilin is observed, are marked in Figure 3. (a) Basal area of the ScA, (b) basal area between the CuA and MP1+2, (c) the area of MJ, (d) the region between MP1+2 and RP3+4, (e) basal area of the MP1+2, (f) basal region of the MB, (g) the region of BZ, (h) basal region between the AA and CuA, (i) basal region between the RP2 and RA4+RP1, (j) the wing membrane in the medical field, (k) the wing membrane in the anal field, (l) the wing membrane at the wing tip.

IFM observation of UV-bands confirmed resilin occurrence in several areas of the hind wings. Auto-fluorescence of resilin was observed in locations that needed to be flexible, such as the MB (Figure 4(f)). Strong fluorescence was also observed in some specific regions (Figure 4(b,c,h,i)), such as the marginal region of the wing veins (Figure 4(a,e,f)) and the connection areas of wing veins and wing membranes (Figure 4(d,g)). As the thickness of the hind wing varies from the wing root to the tip of the wing, which results in a blurred fluorescence image caused by numerous parts being out-of-focus, we only focus on the fluorescence characteristics of resilin patch at the specific locations. The locations at the bending zone and near marginal joint, resilin function is similar to a flexible hinge, which also plays an important role in the torsional deformation of the hind wing. From multiple sets of sample observations, without resilin in the wing membrane regions appeared blue (Figure 4(j,k,l)).

Note that the black area in Figure 4 shows the skeleton of the veins region where UV rays cannot penetrate. Due to the age of the equipment and variable thickness of the hind wing, we could not capture sharp micrographs for larger areas.

### 3.3. Wing unfolding and kinematics of *C. buqueti*

**Figure 5.**
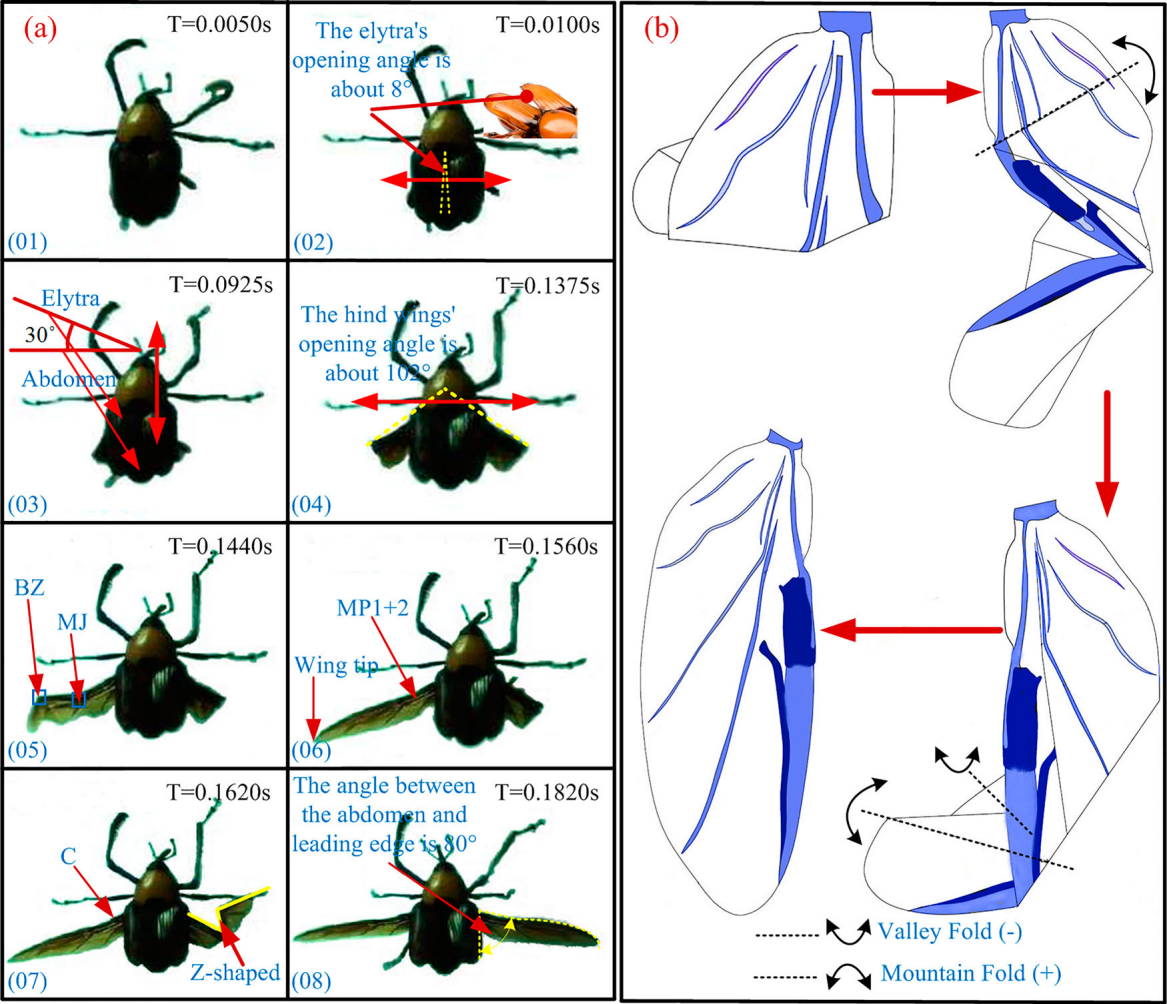
The video sequence of the hind wings of *C. buqueti* unfolding.

The high-speed video recording shows that the unfolding of its all wings (including elytra) totally takes about 0.1820 s. When taking off, the locking mechanism of the *C. buqueti* elytra is unlocked. Then, the elytra began to open to the left and right sides, and in 0.010 s the elytra opened about 8 degrees. Finally, the fore wings moved upward and formed a 30° angle with the horizontal abdomen plane (Figure 5(a 03)). After 0.0925 s, the process of unfolding of the elytra was completed. The hind wings began to unfold after the elytra were completely opened. This process was comparably slow at the beginning because the fore wings were fully unfolded whereas and the hind wings needed to be adjusted from a folded to an unfolded status. After 0.1175 s, the right folded hind wing moved by 51° clockwise and then stopped. After 0.1375 s, the left folded hind wing produced the corresponding counter-clockwise rotation. The left wing immediately tilted down and extended the folded portion. The left wing was fully unfolded after 0.1560 s. It took approximately 0.0210 s for the left hind wing to completely unfold. After 0.1560 s, the right wing tilted down and extended the folded area. The right wing was fully unfolded at 0.1820 s, which took 0.026 s in total. Thus, from a rotation at an angle of 102° (Figure 5(a 04)) to complete unfolding the hind wings took 0.0445 s, accounting for approximately 25% of the entire process.

When the hind wings were fully unfolded, the angle between the leading edge and the abdomen was about 80° (Figure 5(a 08)). The folded hind wings formed three parts, and their area was approximately equal. During the first folding process, the veins RA3 and RP2 at the wing tip were bent along the BZ1 and the center area, respectively (Figure 3). The veins RA4+RP1 and RP2 intersected at the end and the rotation angle along the vein TF1 was approximately 170°. During the second folding process, the veins RA and MP1+2 were in closed proximity so that the angle between them gradually reduced, producing a Z-shaped form of the folded wing. The anal region folded along the anal fold (AF) and was retracted under the main wing area (Figure 5(b)).

### 3.4. The microstructure characteristics of the samples

**Figure 6.**
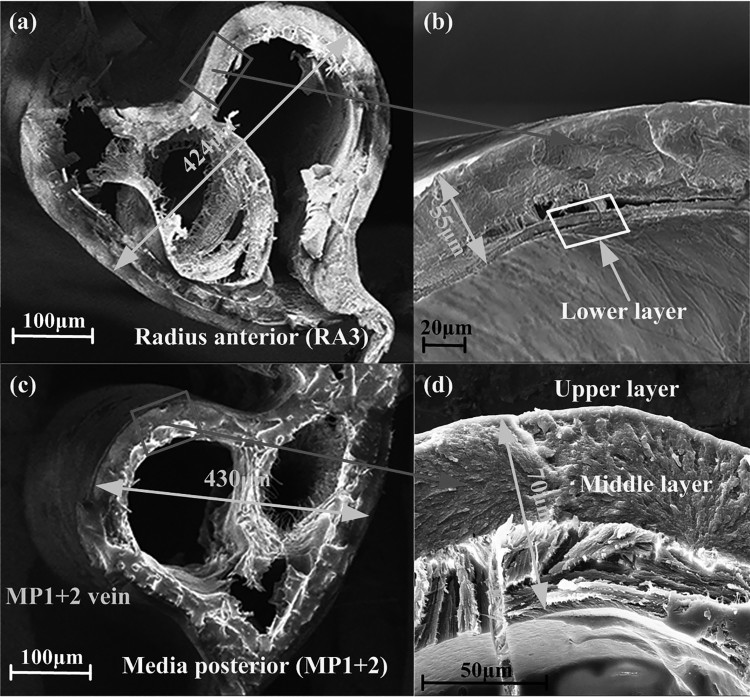
The cross-sectional shape and thickness of the veins of hind wing, which were observed by SEM. (a) The microstructure and thickness of the major RA3, (b) the microstructure details of the RA3 and the wall thickness of hollow structure, (c) the cross-section and average thickness of the major MP1+2, (d) show the microstructure detail of the MP1+2 and the average wall thickness of the hollow structure.

In this work, at the micro level, the SEM observation of the main veins RA and MP1+2 (convex veins) of the hind wings of *C. buqueti* manifest that the wing vein is a kind of anisotropic biological material with the organization of laminar structure. They have a cross-section of a layered structure as shown in Figure 6, and the hollow structure of the veins is visible. The upper and lower surfaces of the veins and the wing membrane both have transverse and longitudinal staggered flocculent fiber layers that are closely connected to each other.

### 3.5. Mechanical properties of selected areas

**Figure 7.**
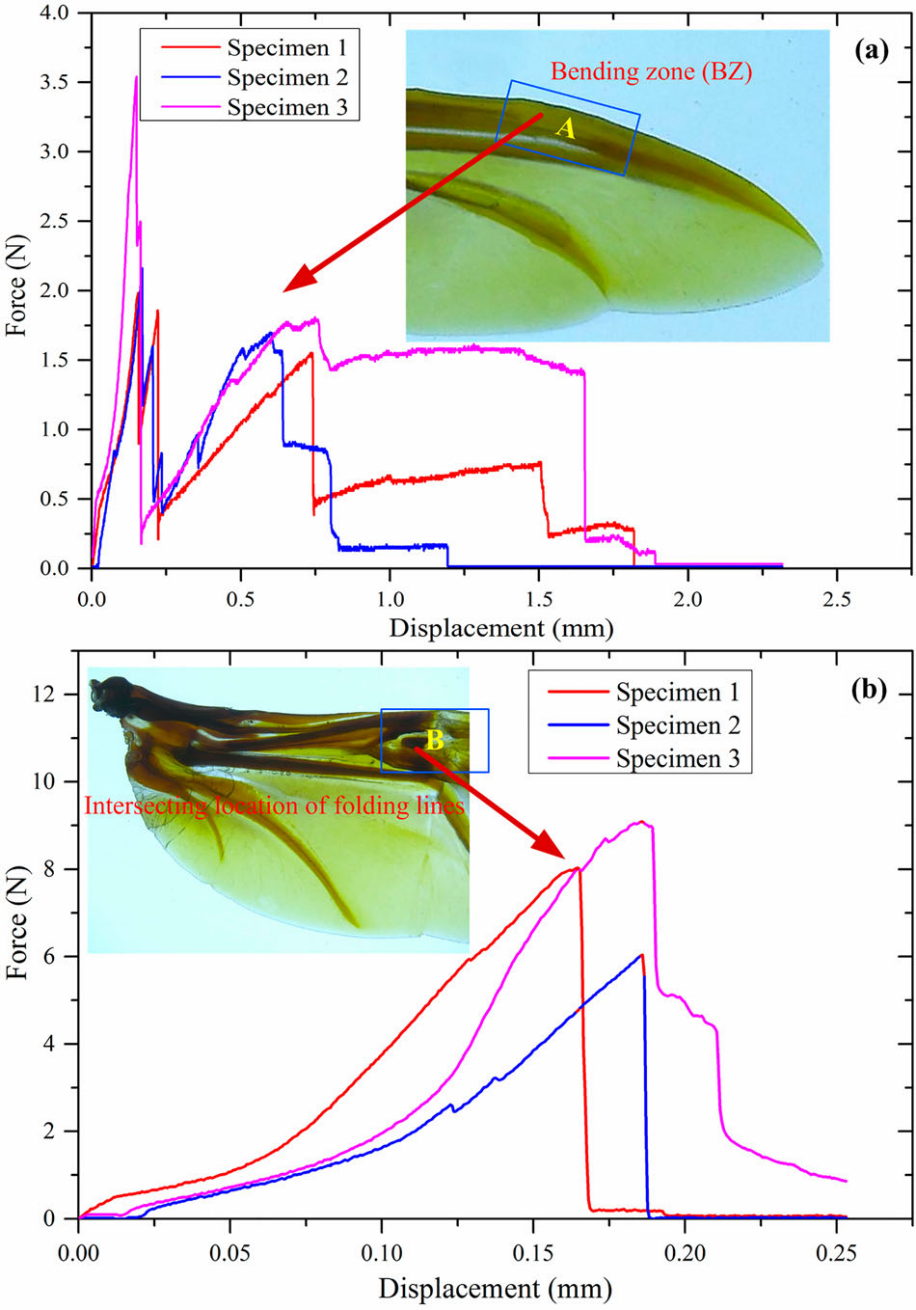
The mechanical properties of the bending zone and the intersection position of folding lines of ‘fresh’ samples.

The mechanical properties of the bending zone and the intersection of folding lines in the hind wings of *C. buqueti* were obtained by multiple sets of tensile testing. The force-displacement curves are shown in Figure 7. It can be seen that in the elastic region, the force versus displacement curves change almost linearly, and the specimen fracture happened after a brief yield deformation. We found that the intersection of folding lines and bending zone can withstand 50–100 times of their own body weight, which was assumed to be 4g for an adult.

## 4. Discussion

The *C. buqueti* is a versatile insects that are a skillful driller and are able to effectively chew in holes and sucks on the bamboo. They can fly freely in dense bamboo forests, which shows that they have evolved special wings to cope with complex conditions. Therefore, from the perspective of biomechanics, the hind wing of *C. buqueti* is not only an outstanding biological material, but also provides an idea for the design of a new concept micro-bionic flapping-wing aircraft. Moreover, the macroscopic and microscopic structures of their hind wings have important guiding significance for the design of lightweight structures and materials.

The previous works (Newman 1982; Gorb 1999; Donoughe et al. 2011) described the morphological characteristics of certain insect wings, and provided important information for a variety of the micro air vehicles research. In this paper, we applied a more systematic approach to describe the basic functional morphology and structural characteristics of the hind wings of *C. buqueti*. The main work includes: (1) fluorescent inverted microscope was used to observe the presence of resilin for its hind wings; (2) the unfolding process of the hind wing was recorded and analyzed by high-speed cameras; (3) microstructural features of the veins were observed using SEM; (4) the force versus displacement curves of the intersection of the folding lines and the bending zone were obtained. The results show that resilin in the hing wings of *C. buqueti* is mainly distributed in the edge of the wing veins and some specific areas (Figure 4), and this protein patches have a strong ability to resist wing's fatigue damage. During the deployment process, the elastic energy stored in the deformation of resilin is released to make the main wing veins RA3 and MP1+2 generate a scissors-like movement.

The cuticle of insects can produce spontaneous fluorescence at wavelengths from blue-green to deep-red. However, our results showed that resilin can also produce spontaneous blue fluorescence of a narrow band at a wavelength of approximately 400 mm (Andersen & Weis-Fogh 1964). Specific wing areas exert a strong spontaneous fluorescence at wavelengths from blue-green to deep-red, such as the hind wings of the *Scarabaeidae*, and *Coccinella septempunctata* (Haas et al. 2000a), the earwig *F. auricularia* (Haas et al. 2000b), the dragonfly *Sympetrum vicinum* (Donoughe et al. 2011), and desert locusts (Hebert et al. 2000). Due to this characteristic, IFM can be used to reveal the desired area of the resilin distributions in insect wings. According to a previous study (Donoughe et al. 2011), the sharpness of small resilin patches in the hind wings of *C. buqueti* (Figure 4) depends on the sensitivity of the inverted fluorescence microscope and on the relative position of the specimen. The detection of specific proteins can be limited because of subtle changes in patch size, leading to deviations regarding the patch projection (Haas et al. 2000a). In order to avoid these artifacts, we examined samples of multiple individuals.

**Figure 8.**
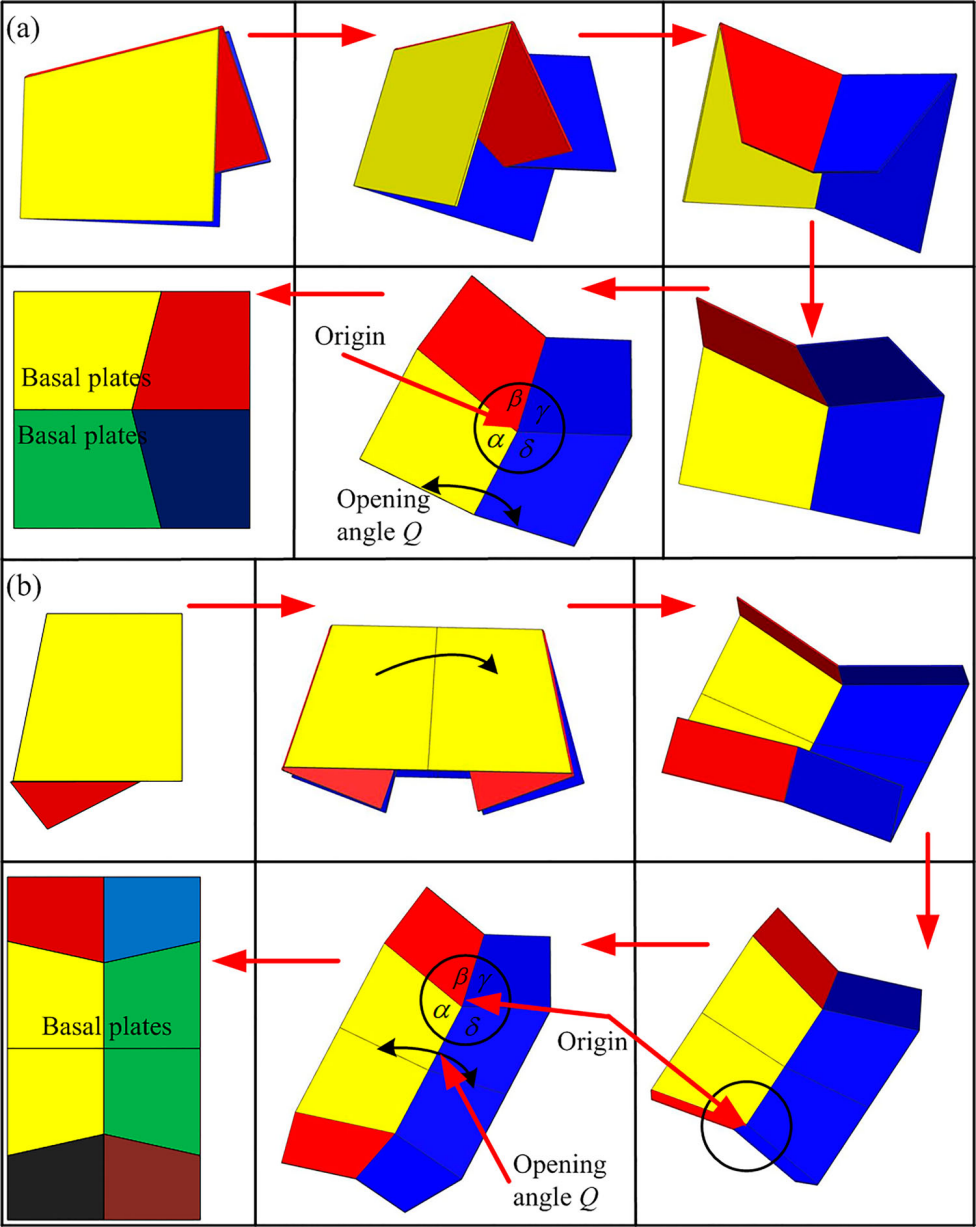
Three-dimensional model of the hind wings folding and unfolding mechanism was modeled. (a) The development process of a four-plate mechanism. (b) The unfolding motion of two four-plate mechanism.

The flying wings unfolding process in *C. buqueti* was recorded using a high-speed camera (Figure 5). The hind wings showed similar mechanisms as those of the cockroach *D. punctata* (Haas & Wootton 1996), which may be seen as a combination of two four-fold planes. This mechanism is folded or unfolded by increasing the opening angle (*Q*) between the two basal planes. In order to form a plane when unfolded (*Q *= 180°), the sum of the four angles at the origin must be 360°. When the opening angle is less than 180°, the produced shape is not plane. When the opening angle exceeded 180°, the structure assumed a position formation mirroring the first stable position (Haas et al. 2000b). Therefore, the sum of the four angles of the wings’ central region (the folding line intersection) was about 350° due to the effect of the branches of the convex–concave veins (Figure 8(b)).

Previous studies confirmed several microstructural properties in insects: Wang et al. (2008) and Chen et al. (2012) revealed that the longitudinal vein of dragonfly wings is a complex sandwich structure with chitin shells and a protein layer containing fibrils. The costal vein of the fore wing of worker honeybee (*Apis mellifera*) is a thick-walled cylinder with one layer (Ma et al. 2017). In contrast, the vein of the hind wing of *C. buqueti* comprised three layers: an upper layer (upper epidermis) which was densely structure, a middle layer, and a lower layer (lower epidermis) which was also densely structured (Figure 6). The middle layer was again a multi-layered structure and was held together from the inside together. The SEM maps produced heart-shaped hollow cross-section shape of the main veins RA3 and MP1+2 (Figure 6), which likely helps to reduce weight and may alleviate the alternating mechanical stress that occurs during flight. The thickness of the main wing veins measured by SEM was 62 and 56 μm, respectively. The middle layer of the wing veins was structured irregularly, and in the fracture process the middle layer of the wing veins showed the characteristics of ductile deformation. At the same time, these flocculent fiber layer structures ensure that the hind wings have lightweight and excellent flexural properties. Recent studies have shown that the design strategy of the laminar structuring as observed in biomaterials can improve the overall mechanical properties of the material such as fracture resistance (Gao 2006; Gupta et al. 2006). Insect wings can, to a large extent, absorb the impact of external energy without structural damage, which may be associated with the observed multi-layer structuring (Vincent & Wegst 2004). Moreover, resilin may affect both flexural stiffness distribution and aerodynamic performance of insect wings. Therefore, we conclude that the multilayer structure of the veins of *C. buqueti* and resilin play an important role for the resistance to torsional deflection and overall elasticity.

The mechanical test of the bending zone and the intersection of folding lines showed that, for the bending zone and the intersection of the fold lines, the samples broke under a specific range of loads. The results showed that the two tested areas were able to withstand mechanical strains of 50–100 times of the insects’ body weight. The mechanical properties of flying wings in may be useful for the design of bionic wings. The flying wings of *C. buqueti* can undergo substantial passive bending deformation by aerodynamic forces without sustaining damage. Moreover, elastic energy stored in resilin may partially contribute to the mechanical properties of hind wings. In the meantime, from the above experiments, it was find that there were a large number of resilin patches in the intersection of folding lines and the bending zone. Therefore, we can determine that the flying wings of *C. buqueti* have strong mechanical properties. This resilin may reduce the alternating stress generated by the wings during flight and folding or unfolding.

## 5. Conclusion

The hind wings of *C. buqueti* need to be relatively large in order to ensure flight capability and so must be folded to completely fit under the fore wing. The Coleoptera evolved a unique solution for unfolding and folding of the hind wings. The preliminary studies on the morphology characteristics and mechanical properties of the hind wing of *C. buqueti* were completed in this paper. We combined four technologies; IFM, high-speed photography, SEM, together with the tensile testing. The microstructures indicated that the hollow structure of the veins can reduce the flight weight of the insect. The function of resilin located in specific areas may reduce the alternating mechanical stress generated by the wings during flight and repeated folding and unfolding. In the past decade, research on resilin has received extensive attention. The mechanical properties of resilin with potentially greater application in various fields. Mechanical tests confirmed superior mechanical properties of the hind wings. Therefore, our results may be of importance for the field of aeronautics regarding the folding and unfolding of aircraft deforming wings, and perhaps for astronautics regarding the folding and unfolding of satellite solar panels.

## 6. Geolocation information

The field of my research involved morphology, lightweight biomaterials, biomimetic structural design and bionic engineering.
